# Chronic Arsenic Exposure and Cardiac Repolarization Abnormalities with QT Interval Prolongation in a Population-based Study

**DOI:** 10.1289/ehp.9686

**Published:** 2007-02-14

**Authors:** Judy L. Mumford, Kegong Wu, Yajuan Xia, Richard Kwok, Zhihui Yang, James Foster, William E. Sanders

**Affiliations:** 1 U.S. Environmental Protection Agency, National Health and Environmental Effects Research Laboratory, Research Triangle Park, North Carolina, USA; 2 Inner Mongolia Center for Endemic Disease Control and Research, Huhhot, Inner Mongolia, China; 3 RTI International, Research Triangle Park, North Carolina, USA; 4 Ba Men Anti-Epidemic Station, Lin He, Inner Mongolia, China; 5 Wake Heart Associates, Raleigh, North Carolina, USA; 6 Department of Medicine and; 7 Center for Environmental Medicine, Asthma and Lung Biology, University of North Carolina, Chapel Hill, North Carolina, USA

**Keywords:** arsenic, cardiac repolarization, cardiovascular effects, drinking water, nail, QT prolongation

## Abstract

**Background:**

Chronic arsenic exposure is associated with cardiovascular abnormalities. Prolongation of the QT (time between initial deflection of QRS complex to the end of T wave) interval and profound repolarization changes on electrocardiogram (ECG) have been reported in patients with acute promyelocytic leukemia treated with arsenic trioxide. This acquired form of long QT syndrome can result in life-threatening arrhythmias.

**Objective:**

The objective of this study was to assess the cardiac effects of arsenic by investigating QT interval alterations in a human population chronically exposed to arsenic.

**Methods:**

Residents in Ba Men, Inner Mongolia, have been chronically exposed to arsenic via consumption of water from artesian wells. A total of 313 Ba Men residents with the mean arsenic exposure of 15 years were divided into three arsenic exposure groups: low (≤ 21 μg/L), medium (100–300 μg/L), and high (430–690 μg/L). ECGs were obtained on all study subjects. The normal range for QTc (corrected QT) interval is 0.33–0.44 sec, and QTc ≥ 0.45 sec was considered to be prolonged.

**Results:**

The prevalence rates of QT prolongation and water arsenic concentrations showed a dose-dependent relationship (*p* = 0.001). The prevalence rates of QTc prolongation were 3.9, 11.1, 20.6% for low, medium, and high arsenic exposure, respectively. QTc prolongation was also associated with sex (*p* < 0.0001) but not age (*p* = 0.486) or smoking (*p* = 0.1018). Females were more susceptible to QT prolongation than males.

**Conclusions:**

We found significant association between chronic arsenic exposure and QT interval prolongation in a human population. QT interval may potentially be useful in the detection of early cardiac arsenic toxicity.

Arsenic is an element found in multiple compounds commonly encountered in the environment. Human exposure to inorganic and organic arsenicals may occur from a variety of sources including occupational, medical, and environmental (Lee and Fraumeni 1969; Watanabe et al. 2004; Westervelt et al. 2001). Whether from medical therapy (as with the use of arsenic trioxide in advanced malignancies) or malicious intent (as in acute poisoning), the cardiac effects of massive acute arsenic exposure are well documented (Hall and Harruff 1989; Little et al. 1990; Westervelt et al. 2001). Patients experiencing arsenic toxicity manifest profound abnormalities in repolarization of the heart, resulting in life-threatening malignant ventricular arrhythmias (Ohnishi et al. 2002; St. Petery et al. 1970). Although chronic arsenic exposure via drinking water with high arsenic concentrations has been associated with coronary disease, stroke, and peripheral vascular disease in humans (Navas-Acien et al. 2005; Wang et al. 2002), little is known about its effects on cardiac electrophysiologic properties in general populations experiencing chronic lower-dose arsenic exposure from environmental contamination.

The primary inorganic arsenic exposure in the human population is through ingestion of contaminated drinking water. Arsenic is widely present in natural waters, in the form of inorganic arsenite (As^III^) and arsenate (As^V^). After consumption, inorganic arsenic is converted to methylated derivatives. Although methylation of arsenic has been commonly considered a mechanism for detoxification, recent studies have shown that methylated trivalent arsenicals are more toxic than inorganic arsenic (DeMarini et al. 2003). There are no appropriate animal models available for investigating health effects of arsenic. In 2001, the U.S. Environmental Protection Agency (EPA) adopted the maximum contaminant level (MCL) of 10 μg/L for arsenic in drinking water (U.S. EPA 2001). Significant uncertainties remain regarding mechanisms by which arsenic exerts a deleterious affect on the cardiovascular system, especially at low exposure levels.

The QT (time between initial deflection of QRS complex to the end of T wave) interval on the electrocardiogram (ECG) represents the duration of ventricular electrical systole including depolarization and repolarization. The QT interval varies with heart rate and is typically expressed in seconds or milliseconds in a corrected form (QTc). Several calculations have been used to correct the measurement based on heart rate, and the most commonly employed is the Bazett formula (Bazett 1920). The upper limit of normal for QTc is 0.44 sec (Surawicz and Knilans 2001). Women are known to have slightly longer average QTc intervals than men (0.41 sec for women vs. 0.39 sec for men) (Chou 1986). Prolongation of the QTc intervals ≥ 0.45 sec is associated with increased risk of arrhythmia and mortality. Wu et al. (2003) reported that the mean of the normal QTc interval for Chinese is 0.41 sec (0.42 sec for females and 0.40 sec for males).

Inner Mongolia is an autonomous region in Northern China. The study site is in the Bayingnormen (Ba Men) region located in Western Inner Mongolia on the Hetao Plain, north of the Huang He River (Figure 1). Before installation of the artesian wells in 1980, Ba Men residents used the water from the large shallow wells containing low levels of arsenic for drinking water. Since 1980, > 300,000 Ba Men residents used artesian wells and have been chronically exposed by drinking arsenic-contaminated water. Arsenic occurs naturally in ground water in Ba Men, in levels ranging from nondetectable to 1.8 mg/L (Ma et al. 1999). The counties in Ba Men most affected by arsenicosis are Lin He, Wu Yuan, and Hangjin Hou (Figure 1). Health effects, including dermal (skin hyperkeratosis, alterations in pigmentation, and cancer), neurologic, cardiovascular, and peripheral vascular diseases have been reported in this region (Ma et al. 1999). Ba Men residents have been exposed to a wide range of arsenic concentrations, and it is possible to assess individual exposure because most households (80%) have their own wells. This region is known for its abundance of agricultural products, including dairy and domestic animals for both commercial and personal consumption. In general, nutrition is not a problem among the residents. In Ba Men, drinking water is the main source of arsenic intake. Chinese investigations on arsenic contamination in Ba Men region have shown that the levels of arsenic in the irrigation or surface water from Huang He River are below the U.S. standard for arsenic in drinking water, ranging from 2 to 9 μg/L (Yu et al. 1995). Therefore, arsenic concentrations in grains, beans, poultry, vegetables, and fruits are low. Occasionally, the residents consume freshwater fish or shrimp, but most residents (≥ 60%) do not (Table 1). Thus, intake from diet in this population is not an important source of arsenic exposure. Because migration in this region is minimal among Ba Men residents, and for the reasons stated above, this population provided good opportunities for assessing cardiovascular effects of chronic arsenic exposure in humans. The objective of the study was to assess the effects of chronic exposure to arsenic on cardiac repolarization by investigating ECG QT intervals in a general population.

## Materials and Methods

### Study subjects

**Figure 1:**
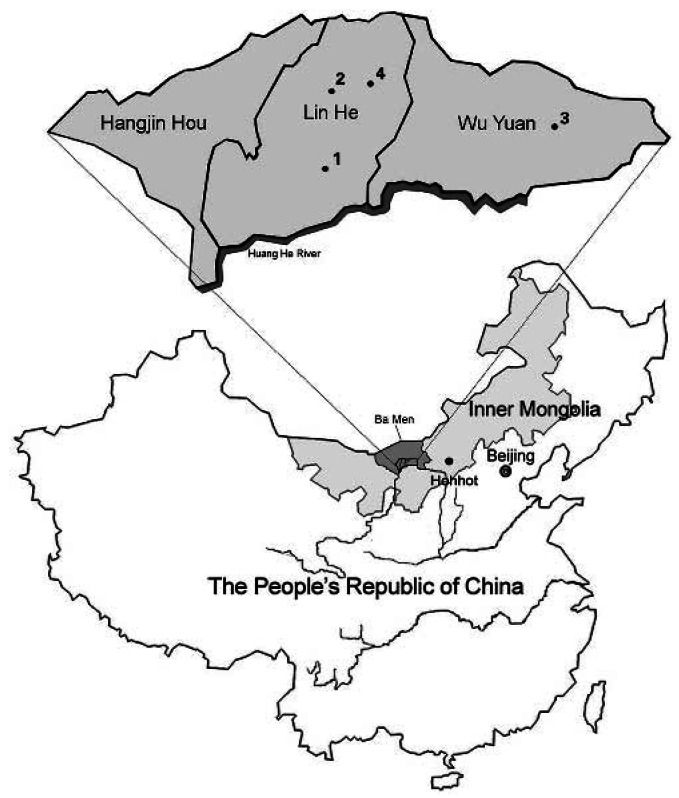
Map of three counties in Ba Men where residents have been chronically exposed to arsenic via drinking water and arsenicosis has been reported. The study sites are located in Long Sheng of Lin He County (1, low exposure), Gu Cheng of Lin He County and Yong Li of Wu Yuan County (2, 3, medium exposure), and Fen Zi Di of Lin He County (4, high exposure).

Because little information is available on the effects of chronic arsenic exposure via drinking water on QT interval in a general population, we conducted a range-finding study. Study subjects had been exposed to a wide range of arsenic levels (low, medium, and high with gaps among the groups), so we investigated at what levels, if any, arsenic exposure affected QT interval in this population. The study subjects included 313 residents of the Ba Men region exposed to different levels of arsenic concentrations via drinking water. One ECG was collected from each subject. Among the 313 ECGs, three manifested right bundle branch block. These subjects were excluded from the data analysis of QT interval. The low-exposed subjects were from the village of Long Sheng in Lin He County, the medium-exposed from Gu Cheng in Lin He County and Yong Li in Wu Yuan County, and the high-exposed were from Fen Zi Di in Lin He County (Figure 1).

We obtained information regarding the consumption of artesian well water with arsenic contamination, tobacco smoking, alcohol consumption, sociodemographic characteristics, and past medical history through questionnaires (Table 1). This study was conducted according to the recommendations of the Declaration of Helsinki (World Medical Association 1989) for international health research. All subjects gave written informed consents to participate in this study. The research protocol met the requirements for protection of human subject certification and was approved by the U.S. EPA.

### Arsenic exposure assessment

#### Water collection and arsenic analysis

Samples of drinking water were collected in acid-washed containers from the study subjects’ homes, as previously described (Mo et al. 2006). Drinking-water samples from the storage tank for well water in each home were collected, stored at −20°C, and transported on ice via air to University of Alberta in Edmonton, Canada, for analysis. Total arsenic was determined using hydride generation atomic fluorescence spectrometry (HGAFS) (Le and Ma 1998). The detection limit for HGAFS is 0.2 μg/L.

#### Nail collection and analysis

Toenail arsenic has been reported to provide an integrated measure of internal arsenic exposure (Karagas et al. 1996). In this study, toenail samples were collected from each study subject as described previously and transported to the U.S. EPA laboratory in Research Triangle Park, North Carolina, for analysis (Schmitt et al. 2005). The nail samples were cleaned by sonification in HPLC-grade water and then acetone washed to remove debris from the nail surface. Toenails were analyzed for arsenic using Instrumental Neutron Activation Analysis (INAA) at North Carolina State University Nuclear Services Department (Raleigh, NC, USA) (Heydorn 1984). The detection limit for INAA is 0.0012 μg/g.

#### QT measurement and evaluation

The ECG QT interval defines the period of ventricular repolarization. The subjects were placed in a supine position. After 10 min of rest, an ECG tracing was recorded for 2 min. In this study, two cardiologists (W.E.S., J.F.) who are certified as electrophysiologists by the American Board of Internal Medicine measured all QT intervals. Discrepancies in the measurement of QT interval between the two cardiologists who evaluated the ECGs were rare. In cases where the measurements were not the same, an average was used. Heart rate of each subject was also determined from the ECG. The physicians were blinded to subjects’ arsenic level as well as the other physician’s interpretation of the ECG. QT interval was corrected for heart rate using the Bazett formula (Bazett 1920). A QTc interval of ≤ 0.44 sec was considered normal.

### Statistical analysis

Arsenic concentration was divided into three categories: low (< 21 μg/L), medium (100–350 μg/L), and high (430–690 μg/L). Age was also divided into three categories: young (9–29 years), middle aged (30–50 years), and older (51–65 years). We assessed exposure by measuring arsenic concentrations in drinking water and nails. QTc interval was considered to be normal if it was ≤ 0.44 sec and abnormal if it was ≥ 0.45 sec. We performed analysis of Spearman correlation coefficients to evaluate the association between arsenic concentrations in drinking water and nails. We compared heart rate and QTc interval measurements in subgroups using Student’s *t*-test. We evaluated bivariate associations between categorical variables using chi-square tests. We used a binary logistic regression model with the QTc interval as dependent outcome and arsenic concentration as the predictor of interest. After prior selection, we used age, sex, smoking status, body mass index (BMI), and pesticide exposure as control covariates in the model. Also two-by-two interactions of the above six main effects are included in the model to control for possible interactions. We used the maximum likelihood estimation method to estimate the logistic model (Hosmer and Lemeshow 2000). The main effects model was fit with all the main effects including age, sex, smoking, BMI, and pesticide exposure. For the final model, we used backward selection with the main effects and all two-way interactions in the full model, eliminating the least significant terms at each iteration and including only the statistically significant variables (*p* < 0.05) in the final model. The final-selected variables maximize the likelihood of distribution of QTc interval outcome and thus significantly predict the QTc interval outcome. All statistical analyses were conducted using SAS version 9.1 (SAS Institute Inc., Cary, NC, USA).

## Results

### Study subjects

Table 1 shows the characteristics of the study subjects. Most of the subjects were farmers with an average of 15 years of chronic arsenic exposure. There were a total of 168 males and 145 females with a mean age of 35 ± 14 years; among these subjects, 33% were smokers. Most subjects had at least a primary education. Diet included frequent meat, fruits, and vegetables. Vitamin use was rare (≤ 2%). Most subjects did not consume seafood from fresh water, or if they did, it was infrequent.

### Water and nail arsenic concentrations

Table 2 shows the nail arsenic concentrations in the low, medium, and high arsenic exposure groups. The mean arsenic concentrations in nails and drinking water in these study subjects showed a positive dose-dependent relationship. Arsenic concentrations in nails and water showed positive correlations in all three groups and also in all subjects.

### QTc and arsenic exposure

**Figure 2:**
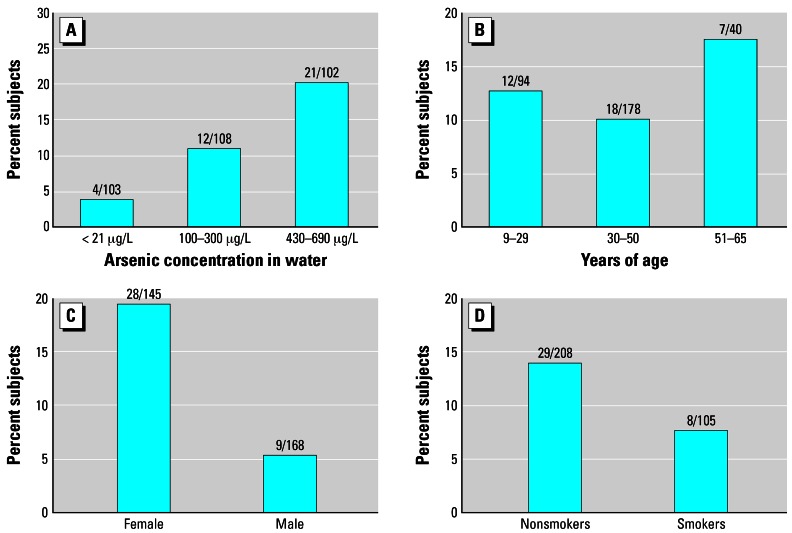
Effects of arsenic exposure (*A; p* = 0.001) and age (*B; p* = 0.486), sex (*C; p* = 0.0001), and smoking (*D; p* = 0.1018) on QTc interval using chi-square tests. The *y*-axis shows percent subjects with QTc interval ≥ 0.45 sec. The numbers above each bar are numerator for the number of subjects with QTc ≥ 0.45 sec and denominator for total number of subjects in this group. *p*-Values are from chi-square tests.

Figure 2 shows the effects of arsenic, age, sex, and smoking on QTc interval. Significant dose-related prevalence rates of QT prolongation were seen in the subjects with increasing water arsenic concentrations (*p* = 0.001) and was most profound in the high-exposure group, with 20.6% of the subjects having marked cardiac repolarization abnormalities. Medium arsenic exposure resulted in 11.1% of subjects having QTc prolongation. The low arsenic exposure group had QTc abnormalities in 3.9% of study subjects. Using chi-square tests, we observed no statistically significant differences in the prevalence of QTc prolongation related to tobacco smoking (*p* = 0.1018) or age (*p* = 0.486). However, there was a significant difference due to sex (*p* = 0.0001). Females were more susceptible to QT interval prolongation than males.

Results from maximum likelihood estimation of logistic regression models, including main effects model and the final model are shown in Table 3. The main effects model controlled for arsenic exposure, age, sex, smoking, BMI, and pesticide exposure; the final model controlled for arsenic exposure, age, sex, BMI, and age–BMI interaction. The results from the main effects model showed that prolonged QTc was associated with arsenic exposure and sex, but not age, smoking, or pesticide exposure. This is consistent with the results from the chi-square tests shown above. Smoke and pesticide exposure were dropped out of the final model. Also dropped out of the final model were all other insignificant interactions except age–BMI interaction, which was statistically significant (*p* < 0.0020). For lower-level BMI, as age increased, the probability of prolonged QTc interval tends to decrease, and for upper-level BMI, as age increased the probability of prolonged QTc interval tends to increase. Both the main effects and the final model showed a dose-dependent relationship in increasing odds ratio (OR) between arsenic concentrations and prolonged QTc intervals (Table 4). In the final model, although all other covariates remain constant, the adjusted OR of prolonged QTc interval for the medium- versus low-exposure group was 3.829 [95% confidence interval (CI), 1.128–12.993] and the high- versus low-exposure group was 8.848 (95% CI, 2.723–28.748). Sex was also a significant predictor, with OR of 5.819 (95% CI, 2.486–13.621) for female versus male.

### Heart rates and QTc

Table 5 shows the comparison of heart rate and QTc interval in the QTc prolonged and normal subjects. The individual withs prolonged QTc demonstrated a statistical significant increase in heart rate in the medium- and high-exposure groups, but not in the low-exposure group. Arsenic has known effects on the nervous system (Hindmarsh et al. 1977). In this study, the subjects with prolonged QTc had slightly higher normal baseline heart rates. This may reflect the arsenic effects on the autonomic nervous system with withdrawal of parasympathetic tone to the sinus node. In Table 5, the mean QTc interval for the low-exposure group was 0.41 sec, which is consistent with the mean of the reported normal QTc for Chinese subjects (Wu et al. 2003). There were statistical differences in QTc measurements between the prolonged QTc and normal individuals.

## Discussion

Chronic arsenic exposure has long been associated with carcinogenesis and atherogenesis, particularly peripheral vascular disease (Simeonova and Luster 2004; Yoshida et al. 2004). Increased mortality from cardiovascular events in individuals in Taiwan who were exposed to arsenic via artesian wells has been reported (Chen et al. 1996). Although Ahmad et al. (2006) reported a study conducted in Bangladesh showing significant difference in QTc interval between the arsenicosis and the non-arsenic–exposed group, cardiac repolarization abnormalities showing dose-dependent relationship with arsenic exposure has not been previously observed. In this study, we evaluated ECG changes in individuals exposed to arsenic via drinking water in Inner Mongolia and assessed the association of prolongation of QTc interval with environmental arsenic exposure. A dose–response relationship was observed, and the prevalence of prolonged QTc interval was significantly associated with arsenic exposure via drinking water in this general population in Inner Mongolia. This is in agreement with the clinical studies showing that arsenic trioxide used in the treatment of acute promyelocytic leukemia induced significant cardiac electrophysiologic abnormalities (QT prolongation) and ventricular arrhythmias (torsade de pointes) (Westervelt et al. 2001). Similar cardiac electrical changes have been shown in acute arsenic poisoning (St. Petery et al. 1970).

The mechanisms by which arsenic induces QT interval prolongation are not completely understood. We hypothesize that this might be caused by the functional alterations in cardiac cell surface channels. Potassium-selective channels play a key role in maintaining the resting membrane potential and repolarization of the action potential in excitable cardiac myocytes (Sanguinetti and Tristani-Firouzi 2006). Hereditary QT prolongation or long QT syndrome occurs in patients with mutations in the cardiac ion channel genes, particularly those involved in potassium ion flow (Keating and Sauguinetti 2001). Among the potassium channel genes, the most commonly observed inherited mutations are found in *hERG* (the human ether-a go-go-related gene) and *KCNQ1* (Curran et al. 1995; Wang et al. 1996). These channelopathies result in significant alterations in cardiac repolarization and predispose the affected individuals to arrhythmias and sudden cardiac death (Sanguinetti and Tristani-Firouzi 2006). *hERG* encodes the voltage-gated potassium channel α subunit underlying I_kr_ (rapidly activating potassium current) (Sanguinetti et al 1995). Potassium current change can result from blockade of the potassium channel, alterations of ion selectivity, or reduction of hERG protein expression. Proarrhythmia is observed in patients treated with drugs that block the hERG channel, including quinidine, dofetilide, antihistamines, and antibiotics (Bednar et al. 2001; Redfern et al. 2003). In addition, the therapeutic use of arsenic trioxide in the treatment of acute promyelocytic leukemia has been hampered by its severe side effects, which include QT prolongation, malignant ventricular tachycardia (torsade de pointes), and sudden death (Unnikrishnan et al. 2001). Recently, arsenic trioxide has been shown to block I_kr_ by inhibition of the processing of the hERG protein in the endoplasmic reticulum (Ficker et al. 2004). In contrast to other drugs, which result in blockade via binding to specific sites within the channel, arsenic trioxide appears to produce hERG liability by inhibition of ion-channel trafficking resulting in reduced channel expression on the cell surface. The QT prolongation seen in our study is most probably the consequence of potassium ion channel alterations induced by arsenic, and may involve hERG trafficking defects.

In this study we observed that age and smoking were not important factors in QT prolongation. However, there was a significant difference related to sex. Women showed an increased sensitivity to toxic effects of arsenic with regard to QT prolongation. Women are known to exhibit average QT intervals slightly longer than those of men and are also more susceptible to the drugs that induce prolonged QT (Drici et al. 1998; Lehmann et al. 1996; Villareal et al. 2001). This may explain the higher frequency of QT interval prolongation in women exposed to arsenic. We are conducting a follow-up study with a larger sample size focusing on women and the sex-specific effects of arsenic as manifested by QT prolongation. Our finding that chronic inorganic arsenic ingestion in a general population can result in QT prolongation may help explain the increased cardiovascular mortality in humans exposed to contaminated drinking water. The risk of significant arrhythmia increases with an increasing QT interval, but is still present with minimal prolongation. In summary, we found significant association between chronic arsenic exposure and QT interval prolongation in a human population, and ECG analysis of the QT interval may be useful in detecting early cardiac arsenic toxicity and in evaluating populations at risk for cardiovascular events. In addition to a larger follow-up study investigating arsenic effects on QT prolongation, we are also conducting a study to investigate cardiac morbidity and mortality in this population. The Chinese government recently has provided assistance in installing water systems in Ba Men to lower the arsenic levels in well water among the Ba Men residents. Reducing arsenic concentrations in drinking water to a safe level in this population would likely eliminate QT prolongation and the associated cardiac risk.

## Figures and Tables

**Table 1:** Study subjects’ information, by low, medium, and high levels of arsenic.

Characteristic	Low (BDL–21 μg/L)	Medium (100–300 μg/L)	High (430–690 μg/L)
No. of subjects	103	108	102
Sex [no. (male/female)]	53/50	57/51	58/44
Age [years (mean ± SD)]	35 ± 14	36 ± 14	34 ± 14
Smoker (%)	29	41	30
Education (%)			
Illiterate	13	19	18
Primary	41	49	42
Middle	39	26	37
High	7	6	3
Water arsenic concentration [μg/L (mean ± SD)]	10.7 ± 6.6	199.9 ± 55.7	568.3 ± 71.1
Water consumption (L/day)	1.6 ± 1.0	1.9 ± 1.1	1.9 ± 1.2
Years of arsenic exposure (mean ± SD)	17.0 ± 3.4	14.6 ± 3.5	14.7 ± 3.3
Pesticide exposure within previous 5 years (% yes)	60	53	50
Diet			
Meat [> 3 times/week (%)]	100	97	100
Fruit/vegetables [> 3 times/week (%)]	100	98	98
Seafood (%)			
Do not eat	60	72	60
≤ 1 time/week	40	28	38
≥ 3 times/week	0	0	2
BMI^a^	22.9 ± 3.7	22.7 ± 3.4	22.6 ± 3.1
Alcohol consumption [> 2 times/week (% yes)]	22	15	12

Abbreviations: BDL, below detection limit; BMI, body mass index.

a BMI was calculated as body weight (kg) divided by squared body height (m^2^).

**Table 2:** Nail arsenic concentrations and correlations between toenails and water arsenic concentrations.

Water arsenic exposure	No.	Nail arsenic concentration [μg/g (mean ± SD)]	Spearman *r*	*p*-Value
All subjects	307^a^	11.80 ± 11.80	0.9056	< 0.0001
Low exposure (< 21 μg/L)	103	1.21 ± 0.74	0.3478	0.0003
Medium exposure (100–300 μg/L)	102	9.79 ± 4.77	0.3547	0.0003
High exposure (430–690 μg/L)	102	24.61 ± 10.65	0.3940	< 0.0001

a Data were missing from six subjects.

**Table 3:** Analysis of maximum likelihood estimates from binary logistic regression models.

Parameter	Estimate	SE	*p-*Value
Main effects model^a^			
Water arsenic (medium vs. low exposure)	1.1184	0.6083	0.0660
Water arsenic (high vs. low exposure)	1.9706	0.5814	0.0007
Age	0.00354	0.0168	0.8328
Sex	1.5678	0.5311	0.0032
Smoking	0.3363	0.5536	0.5436
BMI	−0.0558	0.0652	0.3921
Pesticide exposure	−0.3462	0.4580	0.4498
Constant	−2.9943	1.4464	0.0384
Final model^b^			
Water arsenic (medium vs. low exposure)	1.3426	0.6234	0.0313
Water arsenic (high vs. low exposure)	2.1802	0.6012	0.0003
Age	−0.2524	0.0835	0.0025
Sex	1.7611	0.4340	< 0.0001
BMI	−0.5237	0.1701	0.0021
Age/BMI interaction	0.0123	0.00398	0.0020
Constant	6.0435	3.2398	0.0621

a Main effects model adjusting for arsenic, age, sex, tobacco smoking, BMI, and pesticide exposure.

b Final model adjusting for arsenic, age, sex, BMI, and age/BMI interaction.

**Table 4:** Estimated ORs (95% CIs) for relationships between QTc intervals and arsenic exposure.

Water arsenic exposure	Main effects model^a^	Final model^b^
Low exposure (reference group)	1.0	1.0
Medium vs. low exposure	3.060 (0.929–10.081)	3.829 (1.128–12.993)
High vs. low exposure	7.175 (2.296–22.425)	8.848 (2.723–28.748)

a Main effects model adjusting for arsenic, age, sex, tobacco smoking, BMI, and pesticide exposure.

b Final model adjusting for arsenic, age, sex, BMI, and age/BMI interaction.

**Table 5:** Heart rate and QTc among the subjects with prolonged QTc and normal QTc subjects (mean ± SD).

	Heart rate			QTc		
Water arsenic exposure	Subjects with QTc ≤ 0.44 sec (*n*)	Subjects with QTc ≥ 0.45 sec (*n*)	*p-*Value^a^	Subjects with QTc ≤ 0.44 sec (*n*)	Subjects with QTc ≥ 0.45 sec (*n*)	*p-*Value
Low exposure	66 ± 10 (99)	73 ± 13 (4)	0.1910	0.409 ± 0.021 (99)	0.467 ± 0.016 (4)	< 0.0001
Medium exposure	68 ± 10 (96)	80 ± 15 (12)	0.0194	0.407 ± 0.021 (96)	0.456 ± 0.017 (12)	< 0.0001
High exposure	68 ± 10 (81)	76 ± 10 (21)	0.0018	0.410 ± 0.023 (81)	0.459 ± 0.017 (21)	< 0.0001
All Subjects	68 ± 10 (276)	77 ± 12 (37)	< 0.0001	0.409 ± 0.023 (276)	0.459 ± 0.017 (37)	< 0.0001

a *p-*Values are from *t*-tests.
